# Internal hernia beneath the left external iliac artery after robotic-assisted laparoscopic prostatectomy with extended pelvic lymph node dissection: a case report

**DOI:** 10.1186/s40792-019-0609-6

**Published:** 2019-03-28

**Authors:** Shigeo Ninomiya, Shota Amano, Tadashi Ogawa, Yoshitake Ueda, Norio Shiraishi, Masafumi Inomata, Katsuhiro Shimoda

**Affiliations:** 1 Department of Surgery, Cosmos Hospital, 1131-1 Tomuro, Usuki, 875-0051 Japan; 2 Department of Comprehensive Surgery for Community Medicine, Oita University Faculty of Medicine, 1-1 Idaigaoka, Hasama-machi, Yufu, 879-5593 Japan; 3 Department of Gastroenterological and Pediatric Surgery, Oita University Faculty of Medicine, 1-1 Idaigaoka, Hasama-machi, Yufu, 879-5593 Japan

**Keywords:** Internal hernia, External iliac artery, Extended pelvic lymph node dissection

## Abstract

**Background:**

Formation of an internal hernia beneath a skeletonized pelvic vessel after pelvic lymph node dissection is extremely rare. We report a case of an internal hernia formation beneath the left external iliac artery after a robotic-assisted laparoscopic prostatectomy with extended pelvic lymph node dissection.

**Case presentation:**

A 72-year-old man visited our hospital complaining of severe lower abdominal pain. On physical examinations, his abdomen was distended and tympanitic with rebound tenderness and muscular defense. Abdominal non-enhanced computed tomography showed a small bowel obstruction with marked ascites. A coronal non-enhanced computed tomography image revealed thickened loops of small bowel with surrounding mesenteric edema in the left lower quadrant. Enhanced computed tomography was not performed because we decided to perform urgent surgery with a diagnosis of strangulated small bowel obstruction based on physical examination and the computed tomography findings. The patient underwent urgent laparotomy at which time bloody ascites was seen in the peritoneal cavity. The ileum, which was approximately 60 cm proximal to the ileocecal junction, formed a closed loop beneath the left external iliac artery. The incarcerated ileum, 120 cm in length, appeared non-viable with a color change of the ileum to black. We therefore resected the strangulated ileum for a length of 120 cm and performed a functional end-to-end anastomosis. The orifice beneath the left external iliac artery was about 4 cm in diameter. We did not close the orifice because of the risk of injuring the left iliac artery. The postoperative course was uneventful, and the patient was discharged from our hospital 10 days after surgery. Presently, the patient is doing well 5 months after surgery without recurrent disease.

**Conclusion:**

We report an extremely rare case of internal hernia formation beneath the left external iliac artery after a robotic-assisted laparoscopic prostatectomy with extended pelvic lymphadenectomy. Awareness of such complication and early surgical treatment are important when treating patients with this rare occurrence.

## Background

Pelvic lymph node dissection is a standard procedure in many operative treatments in cancer, such as prostate cancer [1], bladder cancer [2], ovarian cancer [3], and cervical cancer [4]. Also, a Japanese multi-institutional study clarified that extended pelvic lymph node dissection (ePLND) with skeletonization of the external iliac artery improved staging and removed a greater number of metastatic nodes in prostate cancer [5]. The commonly seen complications after ePLND for prostate cancer included ureteral injury, major vascular injury, obturator nerve injury, pelvic lymphocele, deep vein thrombus, and leg edema according to a previous report [5]. Formation of an internal hernia beneath a skeletonized pelvic vessel is an extremely rare complication after ePLND. To our best knowledge, only five cases have been reported previously [6–10]. We report a rare case of an internal hernia forming beneath the left external iliac artery after a robotic-assisted laparoscopic prostatectomy with ePLND and review the literature.

## Case presentation

**Fig. 1:**
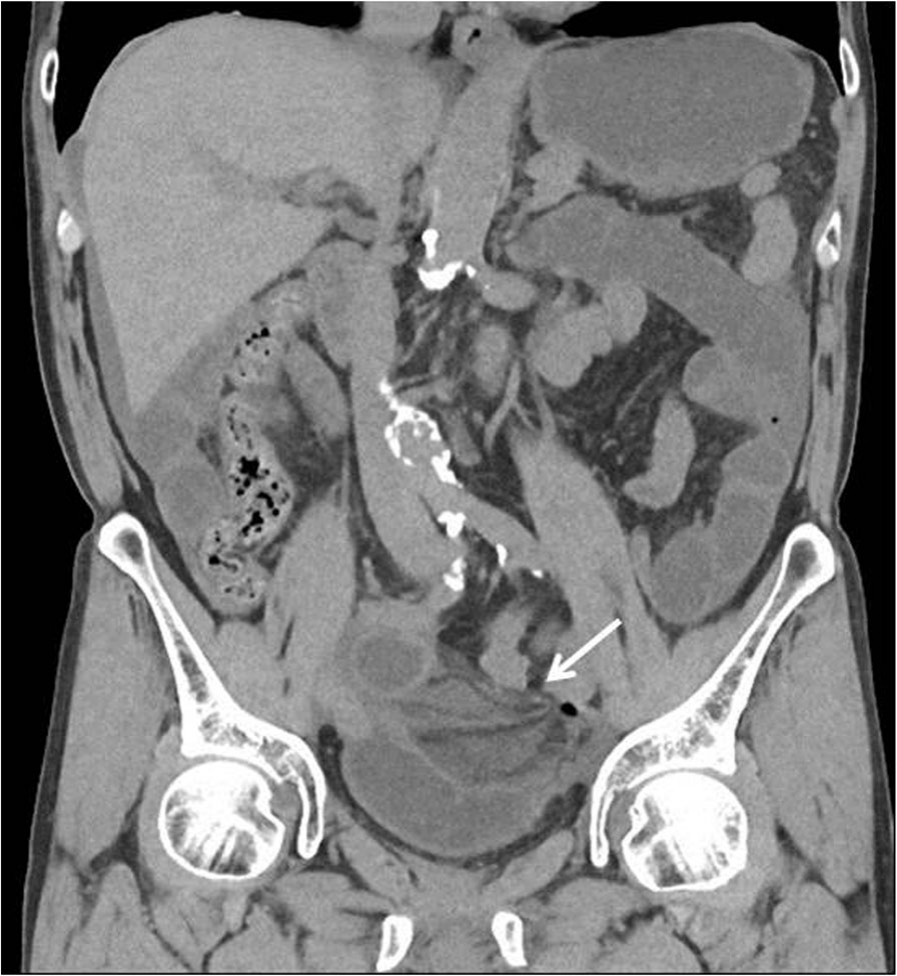
Coronal image of non-contrast computed tomography showed thickened loops of small bowel with surrounding mesenteric edema (arrow) in the left lower quadrant

**Fig. 2:**
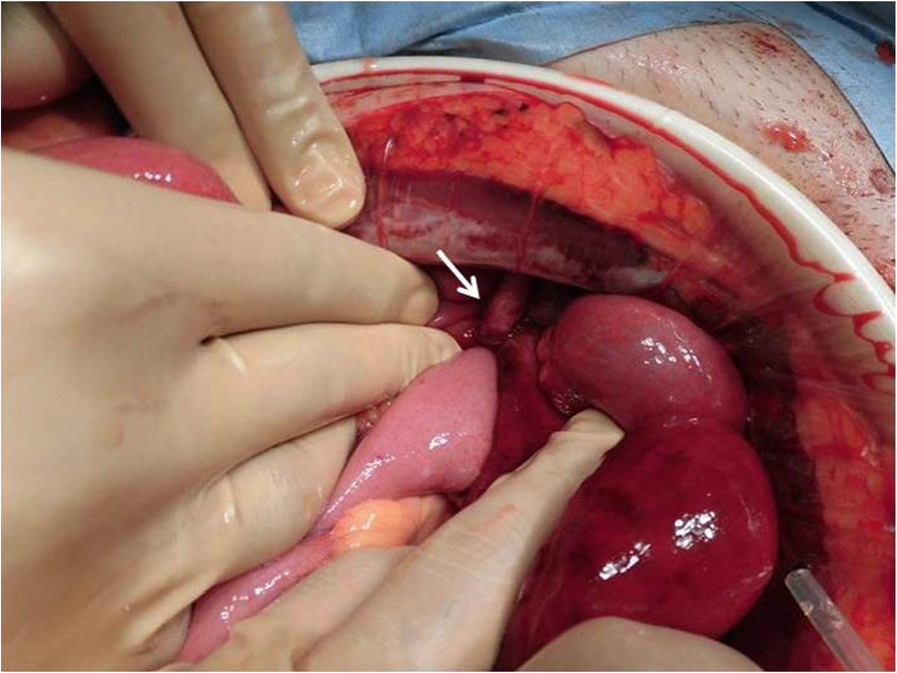
Findings at laparotomy. The ileum, which was approximately 60 cm proximal to the ileocecal junction, formed a closed loop beneath the left external iliac artery (arrow)

**Fig. 3:**
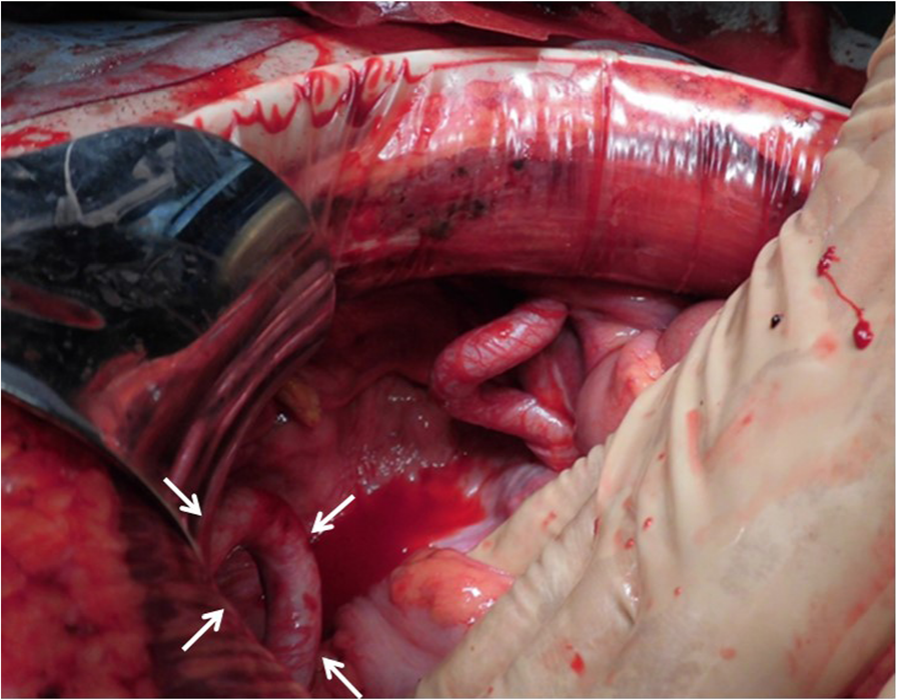
Photograph after the release of the incarcerated ileum. The orifice created by the left external iliac artery (arrow) was left unrepaired

A 72-year-old man visited our hospital with a 1-day history of severe lower abdominal pain, vomiting, and the inability to pass gas or stools. Two months prior to presentation, the patient underwent a robotic-assisted laparoscopic prostatectomy with ePLND for prostate cancer. The patient had no history of previous illness except for prostate cancer and was not taking any regular medications. On physical examination, he showed a pulse of 97 beats/min and blood pressure of 122/64 mmHg. His abdomen was distended and tympanitic with rebound tenderness and muscular defense. Bowel sounds were absent. His groin examination was normal with no signs of herniation through the femoral or inguinal canals. Laboratory data showed only a marked elevation of the white blood cell count (15.0 × 10^3^ μL) and no elevation of his C-reactive protein level (0.01 mg/dL). Abdominal non-enhanced computed tomography (CT) showed a small bowel obstruction (SBO) with marked ascites. Also, a coronal non-contrast CT image revealed thickened loops of small bowel with surrounding mesenteric edema in the left lower quadrant (Fig. 1). Based on these physical and radiological findings, the patient was preoperatively diagnosed as having strangulated SBO. Enhanced CT was not performed because we decided to perform urgent surgery for the patient as soon as possible. The patient thus underwent urgent laparotomy that initially showed bloody ascites in the peritoneal cavity. The ileum, which was approximately 60 cm proximal to the ileocecal junction, formed a closed loop beneath the tortuous and elongated left external iliac artery after ePLND (Fig. 2), and it was also strangulated by this artery. The incarcerated ileum was gently released by a pressing maneuver from the orifice. The released ileum, 120 cm in length, appeared non-viable, showing a color change to black. We therefore resected the strangulated ileum for a length of 120 cm and performed a functional end-to-end anastomosis. The orifice beneath the left external iliac artery was about 4 cm in diameter (Fig. 3). In addition, there was no hernia sac around the artery. We found it risky to try to close the orifice because of the risk of injuring the iliac artery and decided to leave it unrepaired. The postoperative course was uneventful, and the patient was discharged from our hospital 10 days after surgery. Presently, the patient is doing well 5 months after surgery without recurrent disease.

## Discussion

The incidence of SBO after abdominal operations is about 3–5% in a previous report [11]. Also, only around 6% of SBOs are internal hernias with a total incidence of 0.2–0.9% [6]. More than 90% of all internal hernias are caused by natural or artificial orifices, whereas 53% are paraduodenal hernias [6]. Although there were no reports showing an accurate rate of an internal hernia caused by a vascular structure, it is considered to be extremely rare. In contrast, pelvic lymph node dissection has been a standard procedure for patients with prostate cancer [1], bladder cancer [2], ovarian cancer [3], and cervical cancer [4]. ePLND isolates the vessels from neighboring tissues, thus creating a potential hernial defect. However, an internal hernia beneath the external iliac artery after ePLND is an extremely rare complication. To our best knowledge, only five cases of internal hernia beneath the external iliac artery after ePLND have been reported (Table 1). As this condition is a rare but life-threating complication after ePLND, the necessity of retroperitonealization after ePLND should be discussed.

**Table 1:** Reported cases of internal hernia formation beneath the external iliac artery after extended pelvic lymph node dissection (including our case)

Citation	Age (years)	Sex	Diagnosis	Duration (month)^a^
Guba et al. [9]	52	M	Testicular cancer	4
Kim et al. [10]	67	F	Cervical cancer	3
Dumont and Wexels [8]	56	F	Gynecological cancer	48
Viktorin-Baier et al. [6]	51	M	Prostate cancer	12
Kambiz et al. [7]	64	M	Prostate cancer	12
Our case	72	M	Prostate cancer	2

^a^The duration between the initial surgery and the internal hernia

In our case, the internal hernia beneath the external iliac artery occurred 2 months after a robotic-assisted laparoscopic prostatectomy. As shown in Table 1, the median duration between the initial surgery and the internal hernia was 8 (range, 2–48) months. Most cases of internal hernia occurred in the early postoperative period. Although the duration after initial surgery has not been fully elucidated because of the rarity of this complication, careful follow-up after the initial surgery with ePLND is necessary.

Our patient had severe, agonizing abdominal pain. The suspicion of strangulated SBO was our first differential diagnosis according to the physical examination, and it was confirmed with abdominal non-contrast CT. We decided to perform urgent surgery as soon as possible as strangulated SBO is a life-threating disease. Therefore, enhanced CT was not performed. Viktorin-Baier et al. [6] and Dumont and Wexels [8] reported that they could diagnose a patient with an internal hernia beneath the external iliac artery with an enhanced CT examination preoperatively. In general, the diagnosis of internal hernia of the small bowel is a difficult clinical situation. However, the diagnosis of internal hernia beneath the external iliac artery by an enhanced CT examination might be easier than that of other types of internal hernia because it can show the correlation between small intestine and vessels. Also, it might have provided important diagnostic information in our case.

Several treatment options for internal hernia beneath the external iliac artery have been discussed in the previous reports to prevent its recurrence. Guba et al. [9] closed the orifice, including the aorta, by peritoneal graft. Kim et al. [10] also closed the orifice with the peritoneum. Viktorin-Baier et al. [6] reported a case in which performing a resection of 2 cm and end-to-end anastomosis of the elongated artery resulted in shortening of the elongated artery. Kambiz et al. [7] and Dumont and Wexels [8] did not close the orifice because of the risk of injuring the iliac artery. We also decided to leave the orifice unrepaired on the basis of their reports. Another option was to use a mesh to close the orifice. However, we considered that the use of mesh in our patient with a contaminated and dirty strangulated SBO should be avoided to minimize the risk of infection. As so few similar cases have been reported worldwide, the appropriate treatment remains controversial. Therefore, further accumulation of cases and longer follow-up of our patient are needed to determine whether this simple procedure has been sufficient.

With the advances in laparoscopic surgery, Dumont and Wexels [8] reported a case of laparoscopic reduction of incarcerated small bowel beneath the left external iliac artery. Although laparoscopic surgery is reported to be useful for SBO, careful consideration is needed because of the surgical difficulty caused by the limited working space in the abdominal cavity. Date et al. [12] noted that SBO with severe abdominal distension and peritoneal signs suggesting peritonitis might be contraindications for laparoscopic surgery. On the basis of the physical and radiological findings, we selected conventional open surgery for our patient with suspicion of strangulated SBO and peritonitis. Furthermore, it might have been difficult to release the ileum by laparoscopic technique because the ileum was completely incarcerated and strangulated by the orifice beneath the left external iliac artery in our patient.

## Conclusions

In conclusion, internal hernia beneath the external iliac artery is a rare but serious complication after ePLND. Awareness of such complication and early surgical treatment are important in treating patients with this rare occurrence.

## Abbreviations

CT: Computed tomography
ePLND: Extended pelvic lymph node dissection
SBO: Small bowel obstruction
